# The Superior Colliculus Projection Upon the Macaque Inferior Olive

**DOI:** 10.21203/rs.3.rs-2986616/v1

**Published:** 2023-05-30

**Authors:** Paul J. May, Susan Warren, Yoshiko Kojima

**Affiliations:** University of Mississippi Medical Center; University of Mississippi Medical Center; University of Washington

**Keywords:** Oculomotor, Non-human Primate, Saccade Adaptation, Motor Learning, Cerebellum

## Abstract

Saccade accommodation is a productive model for exploring the role of the cerebellum in behavioral plasticity. In this model, the target is moved during the saccade, gradually inducing a change in the saccade vector as the animal adapts. The climbing fiber pathway from the inferior olive provides a visual error signal generated by the superior colliculus that is believed to be crucial for cerebellar adaptation. However, the primate tecto-olivary pathway has only been explored using large injections of the central portion of the superior colliculus. To provide a more detailed picture, we have made injections of anterograde tracers into various regions of the macaque superior colliculus. As shown previously, large central injections primarily label a dense terminal field within the C subdivision at caudal end of the contralateral medial inferior olive. Several, previously unobserved, sites of sparse terminal labeling were noted: bilaterally in the dorsal cap of Kooy and ipsilaterally in C subdivision of the medial inferior olive. Small, physiologically directed, injections into the rostral, small saccade portion of the superior colliculus produced terminal fields in the same regions of the medial inferior olive, but with decreased density. Small injections of the caudal superior colliculus, where large amplitude gaze changes are encoded, again labeled a terminal field located in the same areas. The lack of a topographic pattern within the main tecto-olivary projection suggests that either the precise vector of the visual error is not transmitted to the vermis, or that encoding of this error is via non-topographic means.

## INTRODUCTION

Normal behavior requires movement accuracy. This accuracy must be maintained despite the fact that the body changes both due to growth at early ages and decreased muscle effectiveness later in adulthood. The ability to change motor output in response to sensory feedback indicating an inaccurate movement has been made is termed motor adaptation, and when the inaccuracy is due to an undershooting or overshooting of the target, it is more specifically designated gain adaptation. A common and productive behavioral model for gain adaptation is saccade adaptation (Straube et al., 1997). In this model, the target is moved as the individual is making a saccade. Since visual sensory input is suppressed during a saccade, the perception of the individual is that the saccade over- or under-shot, not that the target moved. In response to repeated trials in which the saccade does not properly obtain the target, the gain of the saccade is changed in an attempt to compensate for missing the target. The same system can be used to demonstrate that the nervous system can also compensate for inaccuracies related to saccade direction (Noto et al., 1999). In order to trigger gain adaptation, the nervous system produces a visual sensory signal indicating the extent of the targeting error, and then uses this error signal to manipulate the gain of the motor output (Wallman & Fuchs, 1998).

The cerebellum is known to play a critical role in the process of saccadic gain adaptation. Optican and Robinson (1980) demonstrated that the central nervous system is able to adapt to surgically weakening of the horizontal recti via tenectomy, but this capacity to adapt is dramatically impaired by ablation of the cerebellum, and more specifically, the oculomotor vermis. Stimulation of the oculomotor vermis produces eye movements in a manner that suggests it is topographically organized (Ron & Robinson, 1973; Noda et al., 1987), although there is no evidence of topography based on the simple and complex spike activity of vermal Purkinje cells (Kojima et al., 2010A; 2010B; Kojima, 2019). Inactivation of the fastigial nucleus, which is a necessary node for output by the oculomotor vermis, largely eliminates saccade adaptation (Robinson et al., 2002; 2006). Pharmacologic manipulation of the oculomotor vermis also affects saccadic motor learning, but seems to mainly involve amplitude increase adaptation (Kojima et al., 2010B; 2011). Recordings from Purkinje cells in the oculomotor vermis show that they changed their complex spiking pattern during the adaptation paradigm (Soetedjo & Fuchs, 2006). Initially, the modulation of complex spike activity in Purkinje cells was thought to just signal the direction, but not the magnitude of the required saccade adaptation (Soetedjo & Fuchs, 2006), but further examination suggested that both direction and size were critical to modulating complex spike activity when a visual sensory error was detected, with most cells tuned to errors of less than 5° (Soetedjo et al., 2008). These changes in complex spike activity are appropriate for driving the adaptive changes in the brainstem motor system that generates saccades (Kojima et al., 2010A).

The complex spikes observed in Purkinje cells are produced by input from climbing fibers that originate in the inferior olive (IO). The source of the visual error signal that drives IO neurons to change the pattern of complex spikes appears to be the superior colliculus (SC). Evidence for this assertion comes from electrical stimulation of the SC (Kaku et al., 2009; Soetedjo et al., 2009) where stimulation of the rostral superior colliculus at levels that do not produce saccades can still produce saccade adaptation. Furthermore, inactivation of the rostral colliculus, which encodes small saccades, severely impairs saccade adaptation (Kojima & Soetedjo, 2018). This is not due to changes in the saccade-related bursts flowing from the SC to the brainstem gaze centers, as the changes in motor activity that occur during saccade adaptation appear to occur downstream of the SC (Melis & van Gisbergen, 1996; Frens & Van Opstal, 1997; Edelman & Goldberg, 2002; Takeichi et al., 2007).

The visual error signal makes its way from the SC to the IO by way of the tecto-olivary projection. This projection is a portion of the crossed predorsal bundle pathway that provides collicular input to the gaze centers in the brainstem and cervical spinal cord (May & Porter, 1992; May, 2006). The main evidence for this pathway in the macaque comes from a study using anterograde transport of tritiated amino acids (Harting, 1977). This study showed an exclusively contralateral projection that terminated within the medial subnucleus of the IO (IOM). However, the animals illustrated for this study had injection sites located in the middle of the SC. As such, they did not indicate whether the tecto-olivary projection is a topographic one, a critical point since saccades of different sizes and directions are mapped in an ordered manner within the intermediate gray layer of the SC. Moreover, they did not include the rostral SC where small visual error signals used to initiate saccade adaptation are produced. A fairly similar terminal distribution of the tecto-olivary projection in the IOM was observed in the cat, although in this species, a sparse ipsilateral projection was present, as well (Weber et al., 1978). More recently, a study of the topography of the collicular projection in the cat has suggested that the rostral SC projects caudally within the IO, while the caudal SC projects rostrally (Kyuhou & Matsuzaki, 1991A). With these points in mind, we set out to investigate the primate tecto-olivary projection by making injections of anterograde tracers into different sectors of the SC using macaque monkeys.

## METHODS

We utilized data from 9 adult and juvenile (2.6–5.0 kg) *Macaca fascicularis* (7) and *Macaca mulata* (2) monkeys in this study. Both male (5) and female (4) monkeys were used, but no sex-specific differences were found. All animals were also used in other, non-conflicting studies. The surgical procedures were approved by the Institutional Animal Care and Use Committees of the University of Mississippi Medical Center and the University of Washington. All animal procedures fell within the guidelines put forth by the Guide for Animal Care and Use issued by the USDA. Animals were sedated before surgery through the use of ketamine HCl (10 mg/kg, IM). They were anesthetized during the surgeries with isoflurane (~ 3%). They also received a preemptive analgesic dose of Carprofen (3 mg/kg, IM). They were given dexamethasone (2.5 mg/kg, IV) to avoid edema and atropine sulfate (0.05 mg/kg, IV) to preclude excess airway secretions. Their temperature was regulated during surgery and vital signs were recorded and kept within normal values. At the conclusion of the surgical procedures, wound edges were infused with Sensorcaine and the animals were provided with Buprenex (0.001 mg/kg, IM) to avoid post-surgical discomfort.

For stereotaxic injections (n = 7) the animals heads were placed in a head holder (Kopf, Tujunga, CA) and a midline incision was made in the scalp. A craniotomy was placed over the midbrain and the cerebral cortex over the SC was aspirated. For biotinylated dextran amine (BDA) (Molecular Probes; ThermoFisher, Waltham, MA) injections (n = 6), a solution of 10% BDA was held in a Hamilton syringe that was angled 22° tip rostral in the rostrocaudal plane. At 2–3 sites in the SC, tracer (0.1–0.2 μl) was injected 1.5 mm beneath the SC surface. For the *Phaseolus vulgaris* leukoagglutinin (PhaL) (Vector Laboratories, Newark, CA) injection (n = 1), a glass micropipette with a ~ 35 μm tip held a 2% solution of the tracer in 0.1M, pH 8.0 phosphate buffer (PB). The micropipette was held at a 22° angle, tip rostral in the rostrocaudal plane. The injection was made at a depth of 1.5 mm beneath the SC surface by passing 7 μA positive current with a 50% duty cycle through the tip with a Midguard iontophoresis unit (Harvard Apparatus, Boston, MA). After injection, the aspiration defect was filled with Gelfoam, the scalp was reapproximated and sutured with vicryl.

For the physiologically localized injections (n = 2), we implanted each monkey with fixtures to prevent head movements, a scleral search coil (Judge et al., 1980) to measure eye position in space, and a recording chamber that was aimed the SC (see Kojima & May, 2021 for details). After the monkeys had recovered from the surgery, we trained them to track a small visual target in a dimly lit, sound-attenuating booth. To prepare for the injection, we plotted the topographic map of the rostral SC (Robinson, 1972; Sparks and Mays, 1980; Munoz and Wurtz, 1995) by recording unit activity and using electrical stimulation. On the day preceding each injection, we made electrode penetrations into the SC to reveal the optimal vector of the chosen locus (Sparks and Mays, 1980; Munoz and Wurtz, 1995; Soetedjo et al., 2002A; 2002B; Kojima and Soetedjo, 2017) by recording and stimulation (50 μA, 500 Hz, 50 ms trains of 0.1 ms cathodal pulses). On the day of the injection, we advanced the tip of the injectrode (a 35-gauge stainless steel tube which was insulated by epoxylite except for its beveled tip to allow electrical stimulation) towards the same site until we heard neuronal bursts related to pseudo-random (in direction and size) target steps and/or the targeting saccades (Kojima and Soetedjo, 2018). We then stimulated to evoke saccades and took the site’s preferred vector as the average vector of 5 evoked saccades. For one monkey, the stimulation evoked a 4.1° saccade in a 337° direction (right and down). For the other monkey, the stimulation evoked a 1.7° saccade in a 144° direction (left and up). In each animal, we injected 120 nl of 10% BDA by using brief pulses of air pressure (PV830 Pneumatic PicoPump, WPI, Sarasota).

Animals survived for 2 (PhaL) to 3 weeks (BDA) following the surgery to allow time for tracer transport. They were once again sedated (ketamine HCl, 10 mg/kg, IM) and then deeply anesthetized (sodium pentobarbital, 50 mg/kg, IP), before being perfused through the heart with a buffered saline rinse followed by a fixative solution containing 1% paraformaldehyde and 1.5% glutaraldehyde in 0.1M, pH 7.2 PB. The brain was blocked in the frontal plane and postfixed for 1 hr in the same fixative solution, then stored in 0.1M, pH7.2 PB at 4° C. Sections were cut at 50 μm (PhaL) or 100 μm (BDA) on a vibratome (Leica VT 1000S, Leica Biosystems, Wetzlar, Germany) and collected serially in 0.1M, pH 7.2 PB. To reveal the BDA, a 1 in 3 series of sections (at a minimum) was incubated overnight at 4° C in an avidin D conjugated horseradish peroxidase (Vector Laboratories, Newark, CA) 1:5000 solution in 0.1M, pH 7.2 PB with 0.05% Triton-X 100. The sections were then reacted with the chromagen diaminobenzidine HCl (DAB) in a 0.1M, pH 7.2 PB 5.0% solution to which 0.05% nickel ammonium sulfate and 0.05% cobalt chloride had been added. The reaction was catalyzed by the addition of 0.011% hydrogen peroxide. To reveal the PhaL, a 1 in 3 series of sections was first pretreated with Triton-X 100 (0.3%) in 0.1M, pH 7.2 PB, then placed in the blocking solution containing 10% normal goat serum. Next, they were placed in a 0.5% solution containing biotinylated goat anti-PhaL (Sigma, St. Louis, MO) overnight at 4°C. The biotinylated anti-PhaL was then tagged using the last solution of an ABC kit (Vector Laboratories, Newark, CA) and the same DAB chromagen procedure as detailed above was used to visualize the reaction product. In all cases, sections were mounted onto gelatinized slides, dried, counterstained with cresyl violet, dehydrated in a graded series of ethanols, cleared in toluene and coverslipped.

For illustration of sections, a Wild M8 stereoscope (Leica Biosystems, Wetzlar, Germany) with a drawing tube was employed. The distribution of terminal label was illustrated using an Olympus BH2 microscope (Olympus Life Sciences, Tokyo, Japan) equipped with a drawing tube. Images of the terminal label were captured on a Nikon Eclipse E600 photomicroscope with a Nikon DXM1200F digital camera by use of Nikon Elements software (Nikon, Tokyo, Japan). Images were adjusted in Adobe Photoshop (Adobe, San Jose, CA) for contrast, color and brightness in order to best match the view seen with the eyepieces.

## RESULTS

The cases analyzed included a variety of SC injection sites that varied both in size and location. In the case of the BDA injections, we examined cases where the injection included much of the SC (n = 2), along with injections that were localized in the rostral (n = 2), middle (n = 2) and caudal (n = 2) SC. Among the cases were two in which the injection site was confined to the lateral SC and one in which it was confined to the medial SC. We will not discuss this point further as we were primarily interested in examining the collicular topography with respect to saccade size, which is mapped rostral to caudal in the SC, and because this medial-lateral variable did not seem to be reflected in substantial differences in the IO terminal field. We will first present a case with a large injection, then cases with more circumscribed injections at mid colliculus, rostral colliculus and caudal colliculus levels. The PhaL injection will be used to illustrate transport from middle levels of the SC.

**Figure 1:**
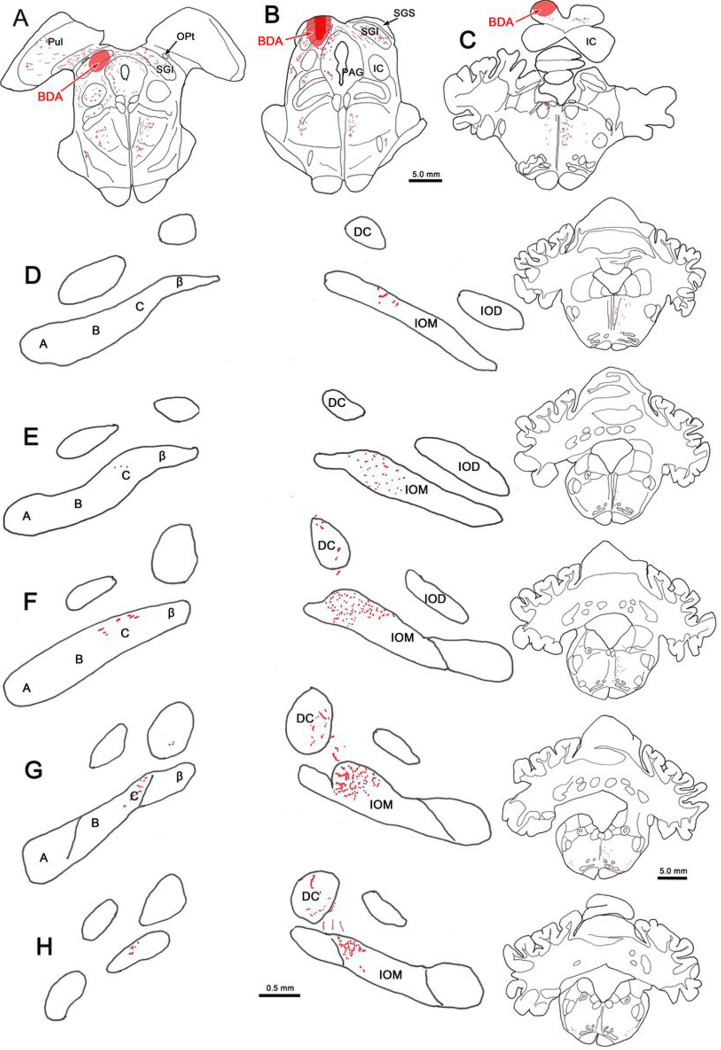
Distribution of BDA labeled tecto-olivary terminal arbors following a large injection in the superior colliculus. **A-C**: The BDA injection involved all collicular layers and extended from the rostral end to the caudal pole of the colliculus. **D-H**: Most of the terminal label (stipple) was located in the C subdivision of the four subdivisions (A, B, C & β) that are located within the contralateral medial inferior olive (IOM). Lesser terminal fields were observed in the contralateral dorsal cap of Kooy (DC) and the C subdivision of the ipsilateral medial inferior olive subnucleus. Just a few labeled terminals were present in the ipsilateral DC. Figure Abbreviations: BDA – biotinylated dextran amine, DC – dorsal cap of Kooy, IC – inferior colliculus, IOD – dorsal inferior olive, IOM – medial inferior olive, MdRF – medullary reticular formation, OPt – olivary pretectal nucleus, P – pyramid, PAG – periaqueductal gray, PhaL – *Phaseolus vulgarus* leukoagglutinin, Pul – pulvinar, SGI – intermediate gray layer, SGS – superficial gray layer

In Fig. 1A–C, a large BDA injection of the SC is illustrated. The injection site extends from the rostral midbrain, where the SC abuts the pretectum to the caudal midbrain, where it lies above the inferior colliculus. While the injection site is centered in the intermediate gray layer (SGI), it extends into substantial portions of the superficial gray layer (SGS) and the deep gray layer (SGP). The outer edge of the injection site extended into the adjacent periaqueductal gray, and it also encroached on the posterior pretectal nucleus (not shown). Labeled axon terminal fields were only observed at the caudal end of the IO (Fig. 1D–H). The densest projection was located within the medial subnucleus of the contralateral IO (IOM). The IOM can be divided into four subdivisions, from lateral to medial: A, B, C and β. It should be noted that while various other approaches to subdividing the nucleus exist, we have utilized that of Barmack (2006). The borders between these subdivisions are poorly defined, so we have mainly identified their relative locations. The vast majority of labeled terminal arbors were located within subdivision C, and filled this division. However, some terminal arbors may have extended into adjacent portions of subdivision B and β. Within the terminal field the densest distribution was located at the very caudal end of the IOM (Fig. 1F–H). An easily seen, but sparse distribution of labeled terminal arbors was also present in the caudal end of the contralateral dorsal cap of Kooy (DC) (Fig. 1F–H). Labeled axons could often be seen running between these two terminal fields. Labeled terminal arbors, albeit a much sparser distribution, were also present ipsilaterally in the IOM (Fig. 1E–H). These were confined to the C subdivision of the subnucleus. Just a very few labeled terminals could be observed within the ipsilateral DC (Fig. 1G).

**Figure 2:**
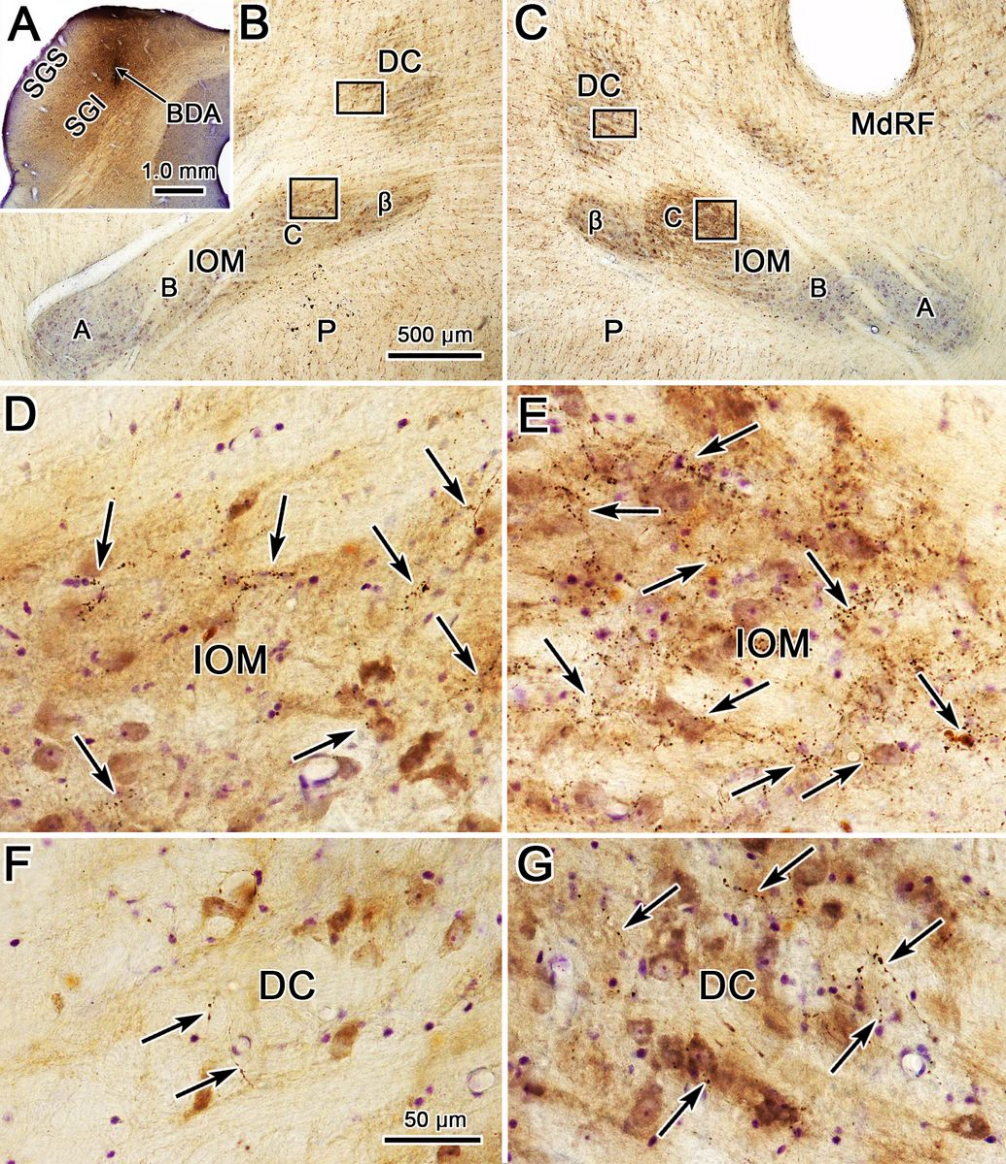
Morphology of BDA labeled tecto-olivary terminal arbors in the case charted in figure 1. **A**. Example section through the BDA injection site in the superior colliculus. **B-C**. Low magnification views showing the A, B, C & β subdivisions within the ipsilateral (**B**) and contralateral (**C**) medial inferior olivary subnucleus (IOM) and the dorsal cap of Kooy (DC). Boxes in **B** indicate areas shown at higher magnifications in **D** and **F**. Boxes in **C** indicate areas shown at higher magnifications in **E** and **G**. BDA labeled axonal arbors (arrows) are most common in the contralateral IOM (**E**). Fewer labeled arbors are present in the contralateral DC (**G**) and ipsilateral IOM (**D**). Only a very few labeled arbors were present in the ipsilateral DC (**F**). Scale in B = C, F = D, E & G

The appearance of the BDA labeled terminal arbors in each of these areas can be directly observed in Fig. 2. The terminal field in the contralateral C subdivision from this injection (Fig. 2A) is dense enough to be visible even at low magnification (Fig. 2C). At higher magnification, the BDA labeled terminal arbors fill the neuropil of this part of the IOM, and appear to be more densely clustered in some regions than others (Fig. 2E). The ipsilateral projection is not intense enough to be visible at low magnification (Fig. 2B), but is easily observed at higher magnification in the C subdivision of IOM (Fig. 2D). This large injection produced quite a few labeled axons in DC on the contralateral side (Fig. 2G). These are not just passing axons, as they display numerous boutonal enlargements. Just an occasional labeled axonal arbor was seen in ipsilateral DC (Fig. 2F), but these axons also displayed boutonal enlargements suggestive of synaptic terminals.

**Figure 3:**
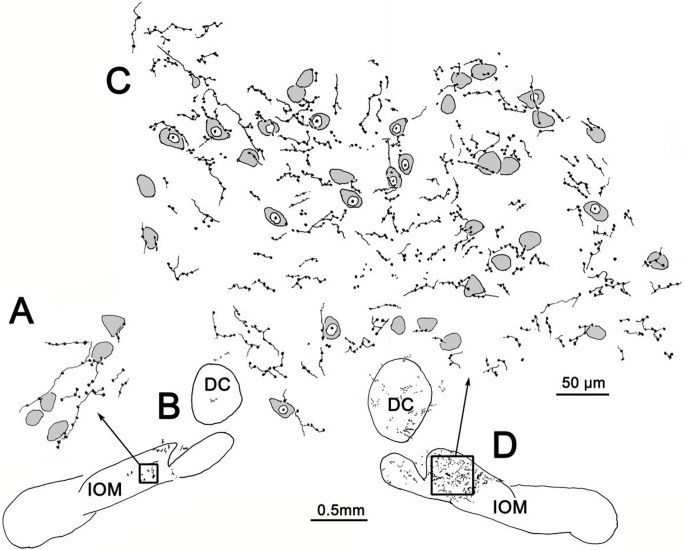
Illustration of BDA labeled axonal arbors in the ipsilateral (**A, B**) and contralateral (**C, D**) medial inferior olive (IOM) from the case illustrated in figure 1. Shading indicates counterstained somata

Figure 3 shows the appearance of labeled axons in IOM drawn at higher magnification. On both the ipsilateral (Fig. 3A–B) and contralateral (Fig. 3C–D) sides, the vast majority of boutons were of the *en passant* variety, although terminal boutons could occasionally be observed at the end of short branches. Relatively few branch points were noted on the axons, however. Some of the boutons were seen to lie in close association with the somata of the counterstained neurons, but most were located in the neuropil, where they presumably contact the dendrites of the same olivary cells.

**Figure 5:**
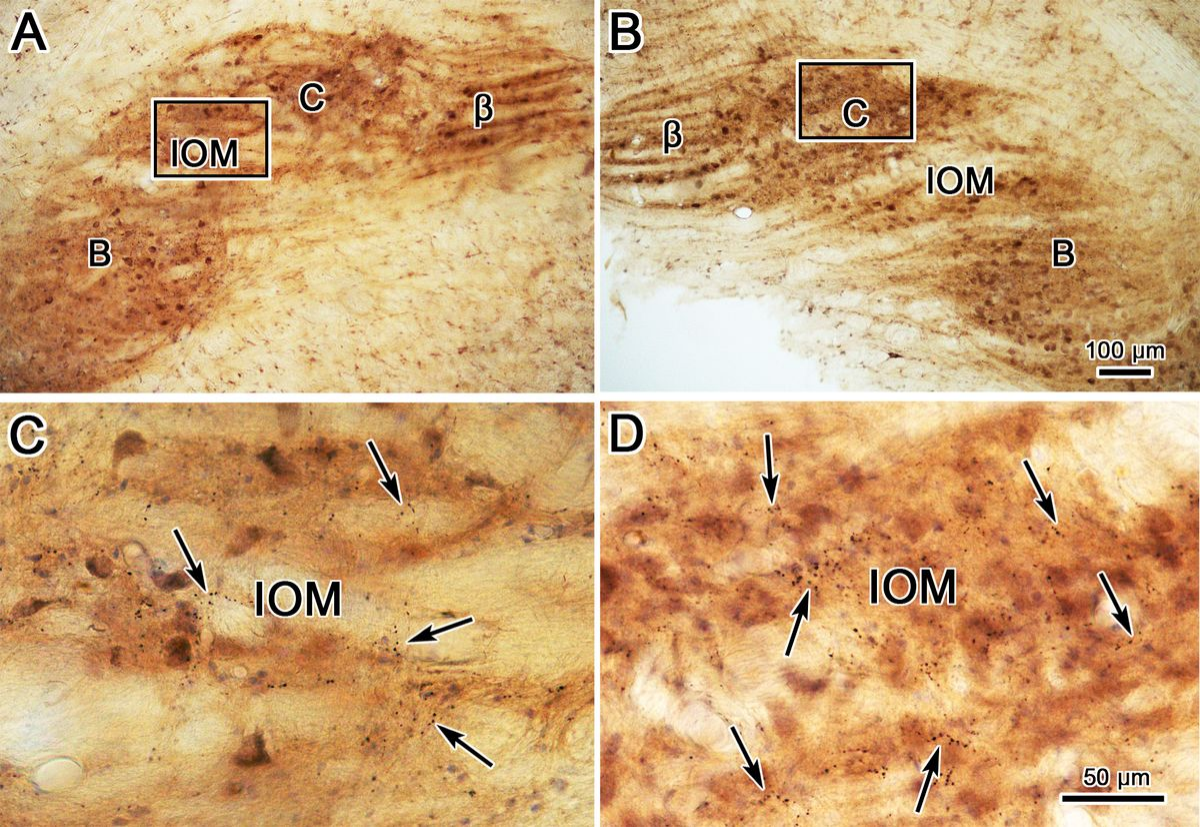
Morphology of PhaL labeled tecto-olivary terminal arbors in the case charted in figure 4. **A-B**. Low magnification views showing the B, C & β subdivisions within the ipsilateral (**A**) and contralateral (**B**) medial inferior olivary subnucleus (IOM). Boxes in **A** and **B** indicate areas of subnucleus C shown at higher magnifications in **C** and **D**, respectivel**y**. PhaL labeled axonal arbors (arrows) are most common in the contralateral IOM (**D**). Fewer labeled arbors are present in the ipsilateral IOM (**C**). Scale in B = A, D = C

An injection of PhaL located at mid collicular levels (Fig. 4E) produced a similar pattern of anterograde labeling. Labeled axonal arbors again produced a dense terminal field in the caudal end of the contralateral IOM that was primarily located in the C subdivision of the subnucleus (Fig. 4A-D). More label was found in the B subdivision of this case than observed in the previous case (Fig. 1D–H). Scattered terminal arbors were also present on the ipsilateral side in the C subdivision of IOM (Fig. 4A-C). In addition, PhaL labeled axons were encountered in the DC, both contralaterally (Fig. 4A-D) and ipsilaterally (Fig. 4A-C). The appearance of the PhaL labeled axons in the IOM is shown in Fig. 5. On both the contralateral side (Fig. 5B,D) and ipsilateral side (Fig. 5A,C) fine axons connect numerous labeled puncta that are primarily located within the neuropil.

**Figure 6:**
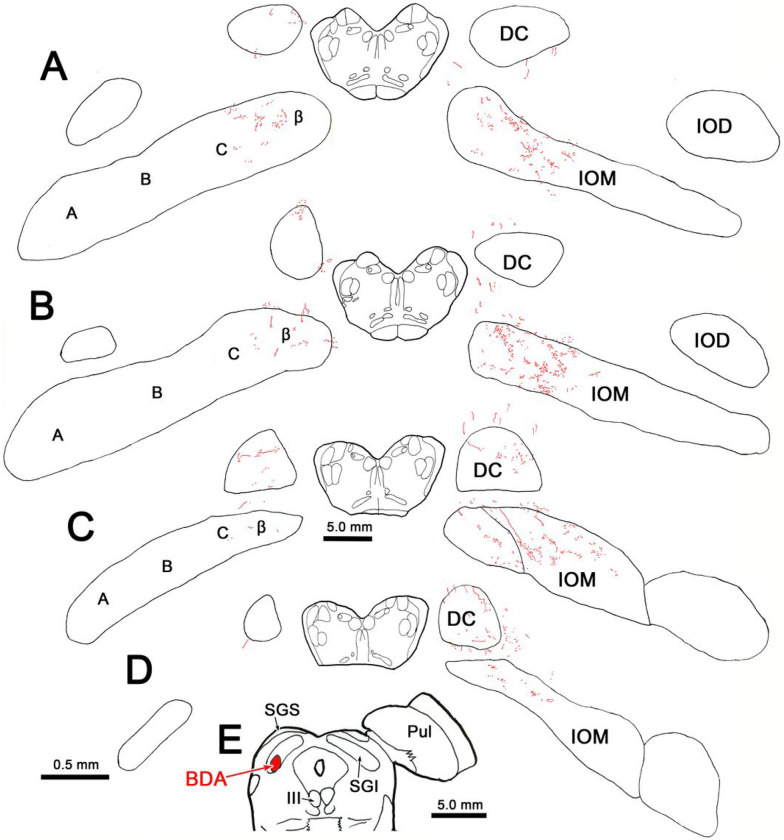
Distribution of BDA labeled tecto-olivary terminal arbors following a small, physiologically guided, injection into the rostral superior colliculus. Electrical stimulation at this location produced a 4.1° saccade. **E**: The BDA injection primarily centered in the intermediate gray layer (SGI). **A-D**: Most of the terminal label (stipple) was located in the C subdivision of the four subdivisions (A, B, C & β) that are located within the contralateral medial inferior olive (IOM), although it extended into adjacent parts of the B and β subdivisions. A sparser terminal field was observed in the C and β subdivision of the ipsilateral medial inferior olive subnucleus. Labeled arbors were also present in the dorsal cap of Kooy (DC), bilaterally

Figure 6E shows a small BDA injection located at a site where electrical stimulation produced a 4.1° saccade to the right and down (at 337°). The pattern of labeling from this injection was similar to that observed with a second case where the injection site produced a 1.7° saccade to the left and up (at 144°). As can be seen here, the BDA injection site was relatively small, and it was confined to SGI at the level shown, with an extension along the micropipette track into superficial gray layer (SGS, not shown). Most of the labeled terminal arbors were found at the caudal end of the IOM on the contralateral side (Fig. 6A–D). The terminal field was densest in the C subdivision of IOM, but extended into both the B and β subdivisions. On the ipsilateral side, sparse labeling of axonal arbors was again found in the C subdivision, but in this rostral injection case more axonal arbors were present in the β subdivision (Fig. 6A–C). Labeled axonal arbors were also present in DC in this case. As with the other cases, more labeled axons were observed contralaterally than ipsilaterally (Fig. 6A–D), but compared to other cases, the rostral injections produced more ipsilateral labeling in the DC.

**Figure 7:**
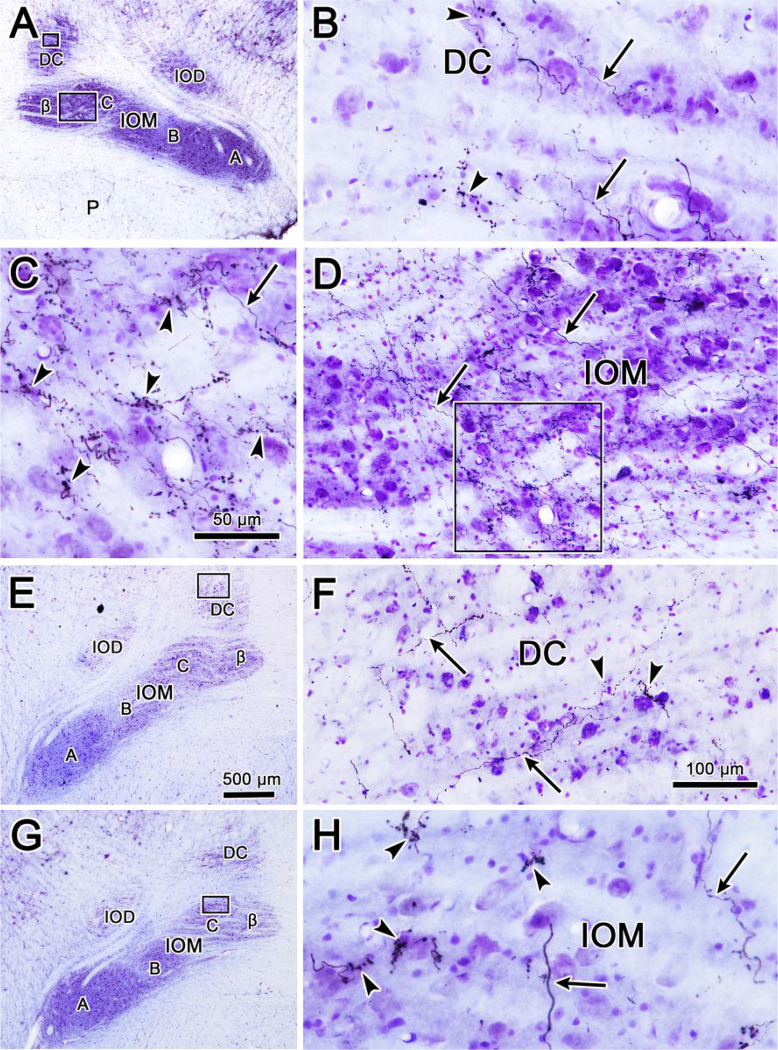
Morphology of BDA labeled tecto-olivary terminal arbors in the case charted in figure 6. **A, E & G**. Low magnification views showing the A, B, C & β subdivisions within the contralateral (**A**) and ipsilateral (**E,G**) medial inferior olivary subnucleus (IOM) and the dorsal cap of Kooy (DC). Boxes in **A** indicate areas shown at higher magnifications in **B** and **D**. Boxes in **E** and **G** indicate areas shown at higher magnifications in **F** and **H**, respectively. BDA labeled axons (arrows) and clusters of labeled boutons (arrowheads) are most common in the contralateral IOM (**D**). The boxed area in **D** is shown at higher magnification in **C**, where clusters of labeled boutons are very evident. Fewer labeled arbors are present in the contralateral DC (**B**), ipsilateral IOM (**F**) and ipsilateral DC (**H**). Scale in E = A & G; F = B & H

The images shown in Fig. 7 reveal the morphology of the labeling produced by this rostral injection. In the contralateral IOM (Fig. 7A,C,D) the labeled axons are studded with numerous *en passant* boutons that tend to be arranged in clusters over the regions of the IOM that are more darkly stained by the cresy1 violet, producing a honeycomb like network. The labeled axonal arbors in the ipsilateral IOM show a similar organization, but there are far fewer of them (Fig. 7G,H). Labeled axons passing through the contralateral DC also produced a number of arborizations with numerous puncta suggesting synaptic contacts (Fig. 7A,B). Similar axons were found on the ipsilateral side of the DC (Fig. 7E,F).

**Figure 8:**
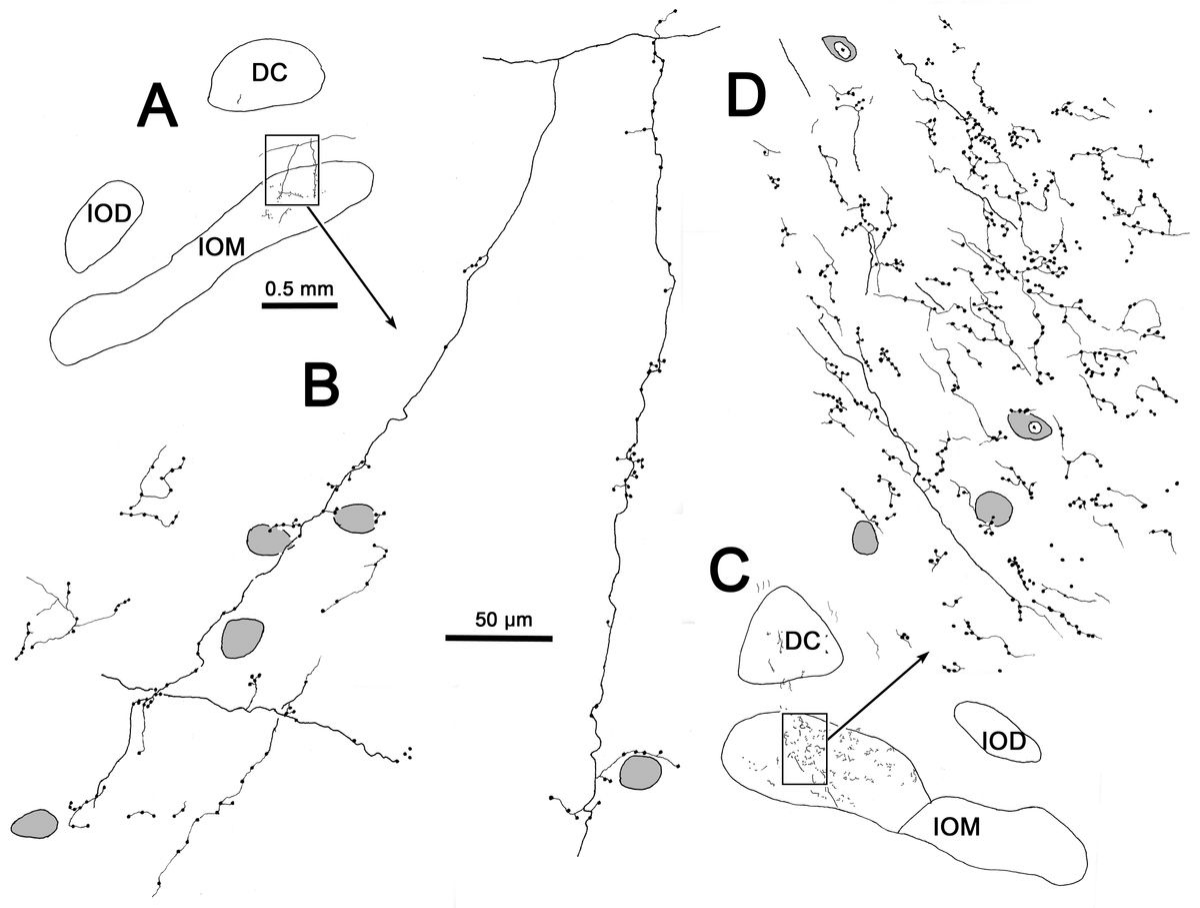
Illustration of BDA labeled axonal arbors in the ipsilateral (**A, B**) and contralateral (**C, D**) medial inferior olive (IOM) from the small rostral injection case illustrated in figure 6. Shading indicates counterstained somata

The excellent level of axonal labeling in this case (Fig. 6–7) allowed detailed drawings of the labeled axons to be made. As shown in Fig. 8D–C, both thicker and thinner labeled axons were present within IOM. The thinner axons exhibited large numbers of *en passant* boutons and occasional terminal boutons on short branches. They tended to be oriented dorsoventrallly. Some of the boutons on these axons are in close association with olivary neuron somata, but the vast majority are located in the neuropil. On the ipsilateral side (Fig. 8A–B), we were able to follow two long axonal branches that were emitted by a larger parent axon. These extended dorsoventrally for long distances into IOM. Within the nucleus, short collateral branches were present. Boutons were found on both the parent and collateral branches.

**Figure 9:**
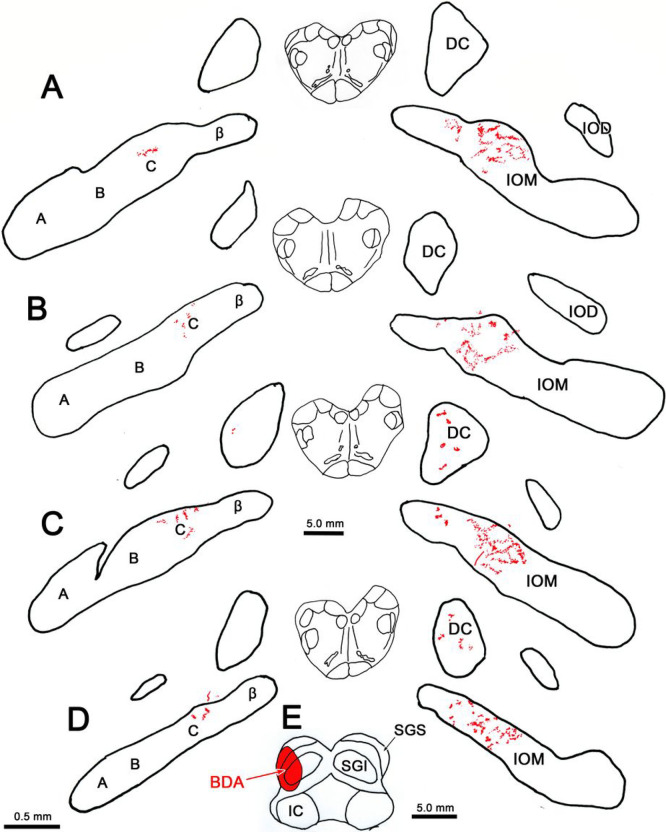
Distribution of BDA labeled tecto-olivary terminal arbors following a small injection in the caudal superior colliculus. **E**: The BDA injection involved all collicular layers and was constrained to the caudal pole of the colliculus. **A-D**: Most of the terminal label (stipple) was located in the C subdivision of the four subdivisions (A, B, C & β) that are located within the contralateral medial inferior olive (IOM). Lesser terminal fields were observed in the contralateral dorsal cap of Kooy (DC) and the C subdivision of the ipsilateral medial inferior olive subnucleus. Just a few labeled terminals were present in the ipsilateral DC (**C**)

An example case of the two with injections constrained to the caudal SC is shown in Fig. 9. The injection site was located in the lateral half of the caudal pole of the SC (Fig. 9E). As with the other injections, the main terminal field was located in the caudal end of the contralateral IOM (Fig. 9A–D). Within IOM, most of the labeled arbors were concentrated in the C subdivision, although a few were located in adjacent parts of the B and β subdivision of the subnucleus. A small number of terminal arbors were located in the C subdivision of the ipsilateral IOM (Fig. 9A–D). In this case, labeled axonal arbors were almost exclusively found contralaterally in DC, at its caudal pole (Fig. 9C–D).

**Figure 10:**
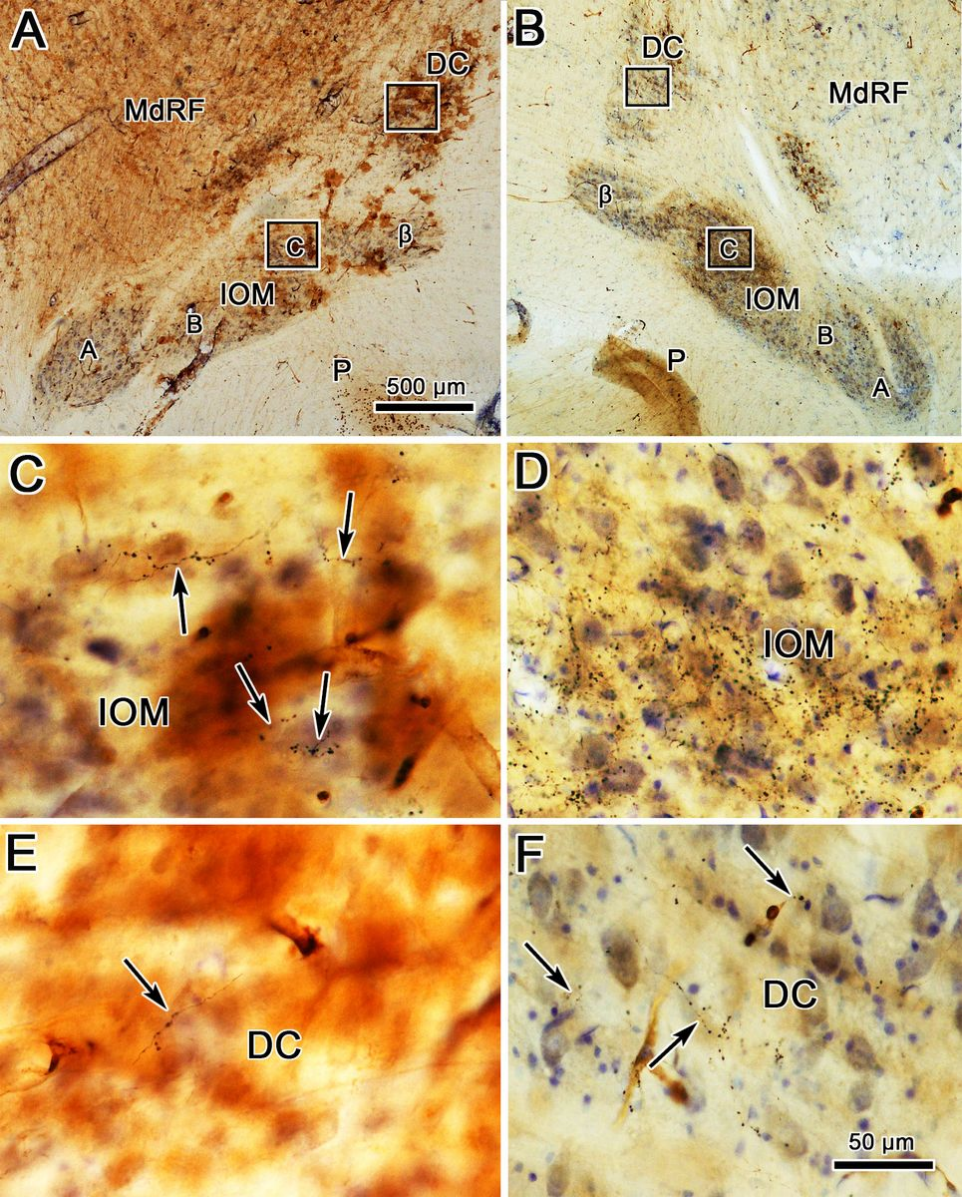
Morphology of BDA labeled tecto-olivary terminal arbors in the case charted in figure 9. **A-B**. Low magnification views showing the A, B, C & β subdivisions within the ipsilateral (**A**) and contralateral (B) medial inferior olivary subnucleus (IOM), along with the dorsal cap of Kooy (DC). Boxes in **A** indicate areas shown at higher magnifications in **C** and **E**. Boxes in **B** indicate areas shown at higher magnifications in **D** and **F**. BDA labeled terminals heavily infiltrate the contralateral IOM (**D**). Fewer labeled arbors (arrows) are present in the contralateral DC (**F**) and ipsilateral IOM (**C**). The arrow in **E** points to the one labeled axonal arbor observed in the ipsilateral DC. Scale in A = B; F = C-E

In the case of the example caudal injection, the labeled terminals were dense enough within the C subdivision of the contralateral IOM that the subdivision was demarcated by the label at low magnification (Fig. 10B). Higher magnification views (Fig. 10D) revealed large numbers of labeled puncta, some in close association with counterstained somata, but most located in the neuropil around the somata. Far fewer labeled axonal arbors were present in the C subdivision of IOM ipsilateral to the injection site (Fig. 10A,C). The DC contralateral to the injection site (Fig. 10B,F) also exhibited a fair number of labeled axons with *en passant* and terminal puncta, albeit far fewer than were present in the IOM. Only one labeled axon with boutons was observed in the ipsilateral DC, as pictured in Fig. 10A,E.

## DISCUSSION

Comparison of the cases with SC injections of anterograde tracers in this study clearly supports the view that the main target of the tecto-olivary projection is the C subdivision at the caudal end of the contralateral IOM. Variable extension of the terminal field into the medially adjacent β subdivision and laterally adjacent B subdivision of the IOM was observed. In addition to this main projection, a much smaller projection to the same region of the ipsilateral IOM was present. The DC also received a sparse input from the tecto-olivary projection at the same levels. This was primarily a contralateral projection. We did not observe any convincing pattern of topographic projection to the IOM based on comparison of injections located in the rostral, middle and caudal SC. However, projections to the ipsilateral DC appeared to increase with more rostral injections. This lack of topography in the tecto-olivary projection of macaques correlates with the evident lack of topographic organization observed in the complex spike activity of primate vermal Purkinje cells (Kojima, 2019).

Neuroanatomical tracers can provide spurious anterograde labeling due to spread outside the target area or uptake by axons passing through the injection site. In the cases we analyzed there was sometimes spread into the periaqueductal gray and pretectum. However, the pattern of termination was largely the same in cases that did not show spread into these structures. To the best of our knowledge, the SC does not generally contain axons that pass through it on the way to the inferior olive. Moreover, the pattern of termination was essentially the same when PhaL was used as a tracer instead of BDA, and PhaL is generally believed to show little axon of passage uptake. Descending axons from the superior colliculus dive directly ventrally, then wrap around the periaqueductal gray on their way to the predorsal bundle (monkey: Moschovakis et al., 1988). Thus, injections at various rostrocaudal levels should not involve axons from neurons located at other levels. In sum, we are confident that the results shown here represent actual labeling from the injection sites.

### Comparison to Previous Studies

The findings presented here are in many ways quite similar to those observed previously. The previous examination of this topic in macaque monkeys showed a terminal field in the same region of the IOM following injection of tritiated amino acids into the middle of the superior colliculus (monkey: Harting, 1977). It should be noted that this paper did not designate a β subdivision, so they describe the terminal field as lying in the B subdivision, instead of the C subdivision. However, their B subdivision is located in the same mediolateral position as the C subdivision of the present study. The lack of reported projections to DC and ipsilateral IOM in the Harting study is most likely due to the limitations of autoradiography for revealing sparse projections. In cats with tritriated amino acid injections of the SC, a terminal field located in the same region of the contralateral IOM was observed (Graham, 1977), with some also observing a sparse projection to the ipsilateral IOM (Weber et al., 1978). This projection and the extent of labeling are very similar to that seen in the present monkey data. Furthermore, that its source lies primarily in the intermediate gray layer of the SC throughout its rostrocaudal extent has been confirmed via retrograde tracing in the cat (Weber et al., 1978; Saint-Cyr & Courville, 1981). The location of the tecto-olivary terminal zone in the rat also appears very similar to that observed for the cat and monkey, although the nucleus containing the terminals is termed the medial accessory olive (Hess, 1982; Akiake, 1992). Only a crossed projection was observed in the rat using tritiated amino acid transport. Thus, the main projection to the contralateral IOM appears to be a conserved mammalian feature. The other projections, ipsilateral to IOM and bilaterally to the DC, may not be found in all mammalian species, or these sparse projections may only be easily observed with more modern tracer techniques. In support of the latter interpretation, a sparse ipsilateral and dense contralateral tecto-olivary projection was observed in hedgehogs by use of BDA (Künzle, 1997), although the much simpler organization of the IO in this species makes more detailed comparison challenging. In bats, however, anterograde transport of wheat germ agglutinin conjugated horseradish peroxidase (WGA-HRP) only revealed a crossed projection to the IO (Covey et al., 1987).

To the best of our knowledge, only one previous study has examined the topography of the tecto-olivary projection. In cats, Kyuhou and Matsuzaki (1991) provided convincing anterograde and retrograde evidence using wheat germ agglutinin conjugated horseradish peroxidase (WGA-HRP) that the rostral SC projects more caudally in the contralateral IOM and the caudal SC projects more rostrally in the contralateral IOM. They also suggested that the medial and lateral SC project contralaterally to the medial and lateral IOM, respectively, although this data was less clear cut. While we saw variations in the contralateral IOM projection between cases in the monkey, we did not observe a pattern of readily apparent differences that appeared dependent on injection site location. This may represent a difference between these two species. Alternatively, WGA-HRP, which tends to more specifically label terminals, may provide a clearer picture of their distribution than BDA that labels the entire axonal arbor. Arguing against the later interpretation is the fact that most of the boutons we observed were *en passant* in nature, so the boutons and arbors should occupy essentially the same territory.

To the best of our knowledge, projections by the SC to the DC have not been previously reported. However, in the figures from the Harting (1977) study in macaques labeled axons are plotted immediately surrounding the contralateral DC. This nucleus is generally believed to be targeted by the nucleus of the optic tract, which lies rostrally adjacent to the SC, and this projection plays an important role in optokinetic eye movements (monkey: Hoffman & Distler, 1989; Fuchs et al., 1992; Mustari et al., 1994; Büttner-Ennever et al., 1996). Furthermore, the nucleus of the optic tract projection to the DC is predominantly ipsilateral, in contradistinction to the predominantly contralateral projection we have observed. The ipsilateral projection was more evident with our more rostral injections, and this difference could be due to involvement of the nucleus of the optic tract, but it was still evident with injections of the middle of the SC that did not reach the pretectum. Thus, we believe we have good evidence of a previously unobserved, sparse projection from the SC to the DC. The role of this projection is currently unknown. However, more recent studies have shown that the SC may be involved in eye movements outside of its cardinal role in saccades. For example, the rostral, but not the caudal, SC shows modulation in neuronal activity with respect to pursuit goals (monkey: Hafed & Krauzlis, 2008). In view of the relationship between pursuit and optokinetic eye movements, the projection we have observed, that comes primarily from the more rostral portion of the SC, may make sense.

### Functional Considerations

During saccadic gain adaptation, a signal indicating the fact the target has been missed is relayed to the vermal portion of the cerebellar cortex via the inferior olive. This is observed as a change in the complex spike activity of Purkinje cells (monkey: Soetedjo & Fuchs, 2006). The complex spikes modulate the simple spike activity in a manner that correlates with the needed change in gain to reduce the target error and properly acquire the target (monkey: Kojima et al., 2010A). Furthermore, these simple spike changes in Purkinje cell activity modulate fastigial nucleus activity, which in turn affects the activity of the brainstem premotor neurons that produce saccades (monkey: Optican & Robinson, 1980; Robinson et al., 2002; Kojima et al., 2008; 2014). There is clear evidence from recording, stimulation and inactivation experiments that the source of the target error signal comes from the SC, by way of the IO (monkey: Fens & Van Opstal, 1997; Kaku et al., 2009; Soetedjo et al., 2009; Kojima & Soetedjo, 2017; 2018). In saccade adaptation paradigms, the target error that initiates the gain change is generally less than 5°. In this study, we have provided direct evidence that the rostral SC, where such small target errors are encoded, projects to the IOM. It is of course this exact tectorecipient part of the IO that projects to the oculomotor vermis (rat: Hess, 1982; Akaike, 1992; cat: Kyuhou & Mitsuzaki, 1991).

The lack of apparent topography in the macaque tecto-olivary projection is surprising in this regard. Considering that saccade size is encoded by the rostrocaudal location of the locus of burst activity in the SC (monkey: Wurtz & Goldberg, 1972; Sparks, 1975), one would presume that target error size following a target miss should be relayed to the vermis by the IO through topographic means. It is possible that the differences in terminal distribution from different loci within the SC are too subtle to be observed within the tight region that is made up of the C subdivision of IOM. These topographic differences are certainly not readily apparent within the paramedian pontine reticular formation, where the topographic map of saccade vectors must be translated into saccade gain appropriate bursts in premotor neurons (monkey: Moschovakis et al., 1998). In the horizontal gaze center, differences in synaptic weighting encode the size of the saccade, not terminal distribution topography (monkey: Grantyn et al., 2002). It is also possible that the more critical information provided by the tecto-olivary-climbing fiber circuit is the fact that a saccade error has been made.

Since all parts of the SC provide input to the IO, and the tecto-olivary projection is a subpart of the general predorsal bundle projection to the brainstem gaze circuitry, it follows that a climbing fiber evoked saccade signal is presented to Purkinje cells before every saccade, regardless of amplitude and direction. What differs about the target error signal for saccade adaptation is the timing; it comes immediately after a saccade has been made, not before, and it does not produce a saccade. (The activity that induces a corrective saccade comes later.) Indeed, it appears that only climbing fiber signals that occur in this window of time after an inaccurate saccade is made, are capable of inducing saccade gain adaptation (monkey: Soetedjo & Fuchs, 2006). Furthermore, any error size will induce adaptation in the correct direction, but not necessarily of the appropriate size (Robinson et al., 1973). On the other hand, adaptation is precisely sensitive to error direction (Kojima & Soetedjo, 2017, Noto et al., 1999). So with respect to amplitude, it is possible that a detailed topographic tecto-olivary projection is not necessary, and that the cerebellum only needs to know whether the saccade was too short or too long (i.e., the direction of the gain error), and does not need to know the precise amplitude of the gain error. Nevertheless, adaptation must ultimately appropriately compensate for the amplitude of the gain error. This may simply occur once the target is achieved by successive small gain changes and the error signal is no longer manufactured.

Another factor to consider is that many SC neurons are of the so-called “open field” type, and do not encode saccade amplitude in their firing rates. Their activity decreases to zero for saccades that are smaller than a unit’s preferred amplitude, but it remains relatively constant for larger saccades (monkey: Takeichi et al., 2007). These characteristics are also found in the complex spikes of some Purkinje cells (monkey: Soetedjo et al., 2008), suggesting there are IO neurons with open field firing characteristics. Topographic projections from the SC would not be needed for a circuit connecting open field SC neurons to the cerebellum by way of the IO. However, a second population of neurons with “closed field” responses is also present in the SC. Similar characteristics have been observed in the complex spikes of some Purkinje cells, so presumably IO neurons that exhibit closed field characteristics are present. In closed field cells, activity quickly decreases for saccades that are smaller, larger or oriented in a different direction than a unit’s preferred amplitude and direction (monkey: Soetedjo et al., 2019). As the neural activity of closed field neurons is sensitive to both the direction and amplitude of the error, one might expect that these IO neurons would receive a topographic projection from the SC and convey it to the cerebellum. It is possible that a topographic tecto-olivary projection by SC closed field neurons is obscured by the fact it is overlain by a non-topographic tecto-olivary projection by open field SC neurons. Alternatively, as noted above, it may be that direction and amplitude specificity are conferred upon IO neurons by non-topographic means. In summary, the lack of topography in the macaque tecto-olivary projection may be understandable, albeit unexpected.

The presence of an ipsilateral tecto-olivary projection to the C subdivision of the IOM was surprising. It is generally assumed that the two sides of the SC work as antagonists, with one side encoding saccades to the right and the other to the left, and that this dichotomy is enforced by inhibitory tectotectal connections (cat: Behan, 1985; Appel & Behan, 1990; monkey: Olivier et al., 1998; Munoz & Istvan, 1998); however, more recent work suggests the interaction between the two sides of the SC is more complex (monkey: Takahashi et al., 2007; 2010; Takahashi, 2019). Perhaps the presence of this sparse projection from the ipsilateral SC represents the anatomical presentation of this complexity. It should also be noted that a sparse projection to the ipsilateral paramedian pontine reticular formation is also observed following the placement of anterograde tracers in the SC (monkey: Moschovakis et al., 1998). The role of these ipsilateral projections remains to be identified.

## Data Availability

The data supporting this study are available for loan upon reasonable request to the authors.
